# Mobile Robotic Platform for Contactless Vital Sign Monitoring

**DOI:** 10.34133/2022/9780497

**Published:** 2022-04-30

**Authors:** Hen-Wei Huang, Jack Chen, Peter R. Chai, Claas Ehmke, Philipp Rupp, Farah Z. Dadabhoy, Annie Feng, Canchen Li, Akhil J. Thomas, Marco da Silva, Edward W. Boyer, Giovanni Traverso

**Affiliations:** ^1^Department of Mechanical Engineering, Massachusetts Institute of Technology, USA; ^2^The Koch Institute of Integrated Cancer Research, Massachusetts Institute of Technology, USA; ^3^Division of Gastroenterology, Brigham and Women's Hospital, Harvard Medical School, USA; ^4^Department of Engineering Science, University of Toronto, Canada; ^5^Department of Emergency Medicine, Brigham and Women's Hospital, Harvard Medical School, USA; ^6^Department of Psychosocial Oncology and Palliative Care, Dana Farber Cancer Institute, USA; ^7^The Fenway Institute, USA; ^8^Boston Dynamics, USA

## Abstract

The COVID-19 pandemic has accelerated methods to facilitate contactless evaluation of patients in hospital settings. By minimizing in-person contact with individuals who may have COVID-19, healthcare workers can prevent disease transmission and conserve personal protective equipment. Obtaining vital signs is a ubiquitous task that is commonly done in person by healthcare workers. To eliminate the need for in-person contact for vital sign measurement in the hospital setting, we developed Dr. Spot, a mobile quadruped robotic system. The system includes IR and RGB cameras for vital sign monitoring and a tablet computer for face-to-face medical interviewing. Dr. Spot is teleoperated by trained clinical staff to simultaneously measure the skin temperature, respiratory rate, and heart rate while maintaining social distancing from patients and without removing their mask. To enable accurate, contactless measurements on a mobile system without a static black body as reference, we propose novel methods for skin temperature compensation and respiratory rate measurement at various distances between the subject and the cameras, up to 5 m. Without compensation, the skin temperature MAE is 1.3°C. Using the proposed compensation method, the skin temperature MAE is reduced to 0.3°C. The respiratory rate method can provide continuous monitoring with a MAE of 1.6 BPM in 30 s or rapid screening with a MAE of 2.1 BPM in 10 s. For the heart rate estimation, our system is able to achieve a MAE less than 8 BPM in 10 s measured in arbitrary indoor light conditions at any distance below 2 m.

## 1. Introduction

The COVID-19 pandemic continues to cause disruption in healthcare systems globally. Despite the presence of COVID-19 pharmacotherapies and vaccines, waves of infection continue to stress healthcare systems globally. While the clinical diagnosis of COVID-19 is not difficult, screening and triaging large numbers of individuals who are infected with COVID-19 poses major challenges among healthcare workers. Part of screening individuals for COVID-19 centers around obtaining vital signs like the heart rate, respiratory rate, skin temperature, blood pressure, and oxygen saturation. Vital sign abnormalities can help clinicians make important disposition decisions, yet the simple act of placing patients on monitors requires personal protective equipment, in-person interactions that may spread disease, and, in settings where resources run scarce, personnel to actually perform vital signs [1]. For these reasons, the development of contactless mobile systems can streamline triage and continuous monitoring in hospitals and in public settings [2].

Previous work has investigated different kinds of contactless monitoring systems using radio signals [3, 4] and radar-based sensors [5]. These systems can easily obtain the respiratory rate (RR) and heart rate (HR) from multiple people without interfering with their daily activity but are unable to capture other vital signs relevant to COVID-19 such as elevated skin temperature and decreased blood oxygenation. In order to screen for fevers from other infectious disease epidemics, commercial infrared (IR) camera systems have been demonstrated to reliably screen individuals for fevers in indoor commercial settings like airports [6]. Similar systems using red-green-blue (RGB) cameras can extract HR [7], blood oxygen saturation [8, 9], and blood pressure [10] from skin RGB pixel changes using a recorded color image of human skin surfaces. This is known as remote photoplethysmography (rPPG) and can be achieved with consumer-level cameras [11]. Recent advances in computer vision (CV) and machine learning enable automatic tracking of the region of interest (ROI) of human faces that are relevant for measuring vital signs, even if the faces are from a crowd and wearing masks. Combining these systems offers the ability to generate contactless vital sign measurements on a mass scale to rapidly detect abnormalities that may be consistent with COVID-19 disease. With an increasing need for solutions to screen individuals who return to work, travel from areas of high viral transmission, and participate in regional- and country-level reopenings during the COVID-19 pandemic, contactless camera systems offer a simple, noninvasive, and scalable system to screen for vital sign abnormalities.

To date, most of these monitoring systems are static and deployed in the emergency department or respiratory clinic triage because of the need to carefully standardize ambient temperature and the distance of the subject to the camera system. In practicality, the chaotic nature of emergency departments which are managing large surges of patients may make these parameters difficult to maintain. Additionally, alternate care areas utilized during large surges of patients may alter the ambient conditions present in an emergency department and render traditional systems inaccurate. There is therefore a need to develop techniques that account for mobility of these systems and their function in disparate environments with changing ambient conditions.

In this work, we developed a mobile robotic system for contactless vital sign monitoring in hospital settings. This system consists of robot-controlled IR and multimonochrome cameras that automatically tracks individuals and measures their skin temperature, HR, and RR while screening for fever, tachypnea, and tachycardia. In order to demonstrate its utility on a mobile system, we deployed the camera system as a payload on the Spot robot (Dr. Spot)—a quadruped robotic system developed by Boston Dynamics [12, 13]—and developed an operator-friendly platform for HCWs. We tested the full system inside a hospital setting to measure vital signs central to COVID-19 evaluation and verified measurements against ground truth sensor readings.

The main contributions of this paper are fourfold. First, we present a fully developed camera system mounted on a mobile robotic system that enables HCWs to easily perform basic triage in nonstandard environments. This system enables HCWs to screen patients while being socially distanced, avoiding disease transmission, and conserving PPE. Second, we propose a novel method for IR camera thermal compensation to enable skin temperature measurement on a mobile, robotic platform. Previous works in machine vision focus on measuring vital signs in static environments, and systems utilizing an IR camera further require the presence of a black body at a fixed distance for thermal calibration [14]. Our proposed thermal compensation method overcomes these challenges by using ambient temperature and the distance to the measured subject to rectify sensor readings. Third, we propose and validate a novel method for measuring the respiratory rate with an IR camera based on periodic temperature variations in a subject's facemask region. This method enables accurate, real-time respiratory rate measurements and is ideal in a hospital setting with mandatory masking requirements. Finally, we demonstrate that the HR can still be obtained from subjects wearing a mask without sacrificing the accuracy. We also evaluate the distance effect on rPPG accuracy and find the minimum threshold in the number of ROI pixels.

## 2. Materials and Methods

### 2.1. Experimental and Technical Design

In response to potential surges of COVID-19 cases, Brigham and Women's Hospital (Boston, MA) deployed a large 25 × 45 foot triage tent (Supplementary Information Appendix I (available here)). Well appearing, ambulatory individuals presenting to the emergency department (ED) with symptoms consistent with COVID-19 disease (upper respiratory infection, fevers, or other exposure to COVID-19) were triaged to the tent for initial evaluation. Participants underwent a brief-nurse-driven interview after which they were seated in the tent waiting area which comprised ten chairs spaced six feet apart. Patients then proceeded to a separate, semiprivate space within the tent, where they met a clinician who conducted a brief, scripted interview regarding COVID-19 exposure and current symptoms. Additionally, the clinician gathered a full set of vital signs (body temperature, HR, RR, and blood pressure) using standard equipment. The clinician then decided if the patient required additional care within the ED or if they can be tested and discharged from the tent.

We developed a robotic platform to enable contactless vital sign monitoring and teleinterviews, thus reducing exposure of HCWs to patients and reducing potential disease transmission. Recruitment of human subjects to test and validate the camera-based system was reviewed and approved by the Mass General Brigham Institutional Review Board (IRB 2021P001334). The robot must operate under several terrains and conditions, including the outdoor triage tent, the indoor ED rooms, and the many indoor-outdoor interfaces. Thus, we collaborated with Boston Dynamics to deploy the quadruped robot Spot (Dr. Spot), which can easily navigate over the loose gravel, curbs, and obstacles in the testing environment [12].

**Figure 1:**
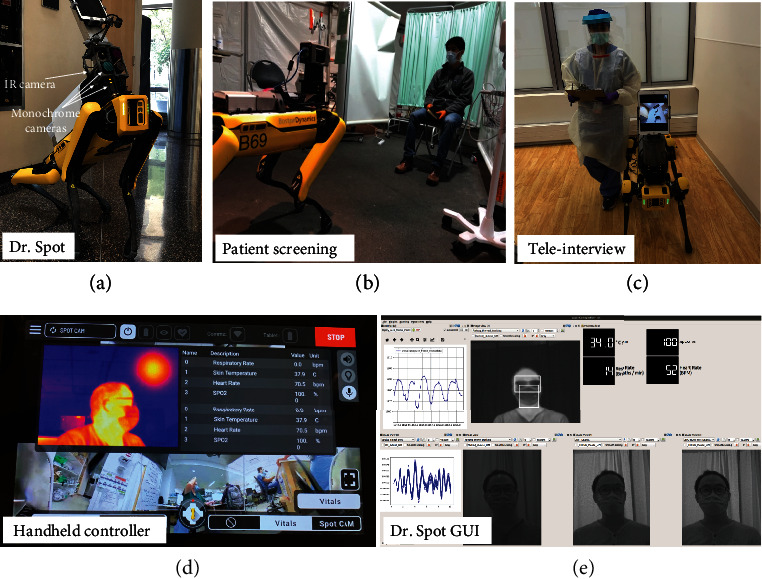
Operation of Dr. Spot. (a) Dr. Spot with the IR camera, three monochrome cameras, and iPad. (b) Patient screening detection with Dr. Spot. (c) Teleinterview with Dr. Spot. (d) Handheld controller for Dr. Spot with vital sign measurement results. (e) GUI for Dr. Spot showing the heart rate waveform, respiratory rate waveform, and estimated skin temperature.

Figures 1(a) and 1(b) show patient screening with Dr. Spot. The IR camera (Optris PI 640i) is used to determine the skin temperature and respiratory rate. The three monochrome cameras have optical filters for wavelengths of 630 nm, 532 nm, and 465 nm; these cameras are used to determine the heart rate.  Figure 1(c) shows teleinterviewing with Dr. Spot. The iPad enables clinicians to interview patients via secure video conferencing. Two graphical interfaces were developed to provide real-time feedback to HCWs.  Figure 1(d) shows the handheld controller used by trained HCWs to control Dr. Spot, while Figure 1(e) shows the more detailed robotic operation system (ROS) GUI.

**Figure 2:**
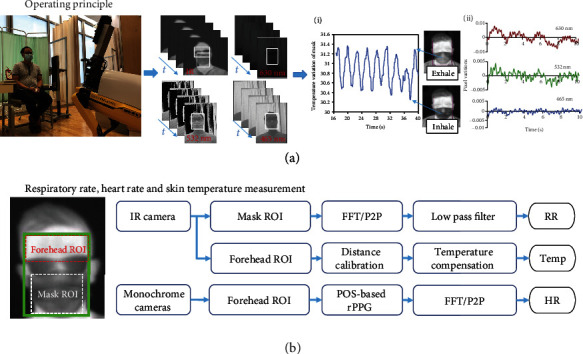
System overview. (a) Operating principle of Dr. Spot showing IR images and RGB monochrome images. (a-i) IR camera waveform for respiratory rate measurement. (a-ii) RGB monochrome waveform for heart rate measurement. (b) Methodology used to calculate vital signs.

The operating principles of the system are shown in Figure 2(a). A HCW maneuvers Dr. Spot in front of a seated patient. The procedures of generating RR, elevated skin temperature, and HR are described in Figure 2(b). There are two regions of interest (ROI) in a subject's face: the forehead ROI is used to measure the skin temperature and HR, while the mask ROI is used to measure RR. To accurately segment the forehead, we employed the InsightFace face analysis library to detect faces and facial landmarks [15]. Since InsightFace is trained on RGB images, we rescaled the raw thermal frames to an 8-bit depth with the corresponding range of [0,255] on each RGB channel. Let *x*_box_, *y*_box_, *w*_box_, and *h*_box_ be the top left *x* coordinate, top left *y* coordinate, width, and height of the facial bounding box, respectively; let *y*_eye_ be the *y* coordinate of the facial landmarks corresponding the eyes. The forehead ROI is selected as the rectangular region with the top-left corner (*x*_box_, *y*_box_) and bottom-right corner (*x*_box_ + *w*_box_, *y*_eye_). The mask ROI is selected as the rectangular region with the top-left corner (*x*_box_ + 0.1∗*w*_box_, *y*_box_ + 0.5∗*h*_box_) and bottom-right corner (*x*_box_ + 0.9∗*w*_box_, *y*_box_ + *h*_box_).

### 2.2. IR Camera: Thermal Compensation, Skin Temperature Measurement, and Fever Detection

Infrared thermography can detect elevated skin temperature which may indicate the presence of a fever. An IR camera measures the skin temperature distribution but is sensitive to both the ambient temperature and the distance to the subject. In the initial iteration of the camera system, we followed suggestions by the IR camera manufacturer FLIR and established a baseline by scanning and saving readings from ten known healthy individuals coming from similar ambient conditions [16]. Readings from future subjects at the same ambient temperature scanned would be compared to this population baseline. Subjects with facial skin temperature higher than the baseline would be asked to undergo further diagnostic evaluation.

The success of this approach relies on a calibrated temperature reference or “black body” that needs to be placed at the same distance from the camera for every measurement. To remove the need of a black body for Dr. Spot, we investigated the effect of ambient temperature and distance to subject on IR camera readings. The distance to subject was acquired after determining the relationship between distance to subject and the face detection bounding box size. Using this relationship, we were able to use face bounding boxes to calculate the distance to the subject. Based on these experimental results, we proposed a thermal compensation algorithm that corrects for the ambient temperature and distance to the subject. We verified the compensation algorithm by comparing compensated temperatures against ground truth sensor readings.

### 2.3. IR Camera: Respiratory Rate Measurement and Tachypnea Detection

Respiration results in heat exchange with the environment; Parsons provides the following equation for estimating this exchange [17]:

(1) $$\begin{array}{c} C_{\text{res}}+E_{\text{res}}=\left(0.0014\;M\left[34-T_{\text{ambient}}\right]+0.0173M\;\left[5.87-P_{a}\right]\right), \end{array}$$

where *C*_res_ is the rate of convective heat loss from respiration, *E*_res_ is the rate of evaporative heat loss from radiation, *M*is the rate of metabolic energy production, *T*_ambient_ is the ambient temperature, and *P*_*a*_ is the ambient pressure. Dr. Spot is mounted with the Bosch BME280 sensor to determine *T*_ambient_ and *P*_*a*_.

The convective heat loss *C*_res_ occurs due to the exhalation of hotter air at body temperature and the inhalation of colder air at ambient temperature. The evaporative heat loss *E*_res_ occurs due to exhalation of air with higher moisture saturation. During normal breathing, heat exchanged with the environment quickly dissipates. However, wearing facemasks creates a “microenvironment” that constrains the breathing environment; the facemask reduces the permeability of air and vapor, limits heat exchange with the ambient environment, and results in heat retention [18]. Inhalation of the warm air retained in the facemask results in a heat transfer from the microenvironment back to the mask wearer.

The thermodynamics of the facemask during respiration suggests periodic temperature variations in the mask ROI corresponding to inhalation and exhalation. We experimentally verify this temperature variation in IR images and propose a novel method to calculate the respiratory rate and to screen for tachypnea (respiratory rate greater than 20 BPM). This variation in temperature is the raw breathing signal. To obtain RR, we proposed and compared two methods. In method one, we computed the average peak-to-peak value and converted this directly into beats per minute. In method two, we apply a low-pass filter to remove the high-frequency band noise followed by a fast Fourier transform (FFT). RR is obtained by selecting the frequency which corresponds to the highest amplitude in the frequency spectrum. The proposed methods were tested experimentally and validated against ground truth sensor readings.

### 2.4. RGB Monochrome Cameras: Heart Rate Measurement and Tachycardia Detection

Remote photoplethysmography (rPPG) is a simple yet low-cost optical technique that can be used to measure blood volume changes underneath the facial skin via a consumer-level camera. These changes can be processed to determine the heart rate and screen for tachycardia (heart rate over 100 BPM). rPPG analysis and characterization are performed on a combination of recorded subjects and the UBFC-rPPG dataset (which comprises of 42 videos with subjects and their ground truth HR) [19]. Characterizations requiring skin segmentation use color-based methods [20].

The light absorption characteristics of bloodstream hemoglobin exhibit a strong peak at the wavelength between 500 and 600 nm, which corresponds to the frequency band of the green light signal captured by an RGB camera. In the HR estimation algorithm, the light absorption characteristics are obtained from the forehead ROI. Previously, we used the filtered wavelengths at 660 nm, 810 nm, and 880 nm [21], which is more motion robust for rPPG in a dark environment, broadening the potential applications of the camera system [22]. In this work, we used three monochrome cameras with filtered wavelengths at 630 nm, 532 nm, and 465 nm in an indoor environment with bright lighting condition.

Normally, rPPG is very sensitive to the presence of motion and noise artifacts. To enable a motion robust rPPG, de Haan and Van Leest presented the POS method [23]. This method superposes the averaged RGB signals into two orthogonal signals *S*(*t*) from which the eventual pulse signal *h*(*t*) is determined. The former is defined as

(2) $$\begin{array}{c} S\left(t\right)=\left[\begin{array}{c} S_{1}\left(t\right)\\ S_{2}\left(t\right) \end{array}\right]=P∙C_{n}\left(t\right)\approx I_{0}∙P∙N∙\left(u_{s}∙s\left(t\right)+u_{p}∙p\left(t\right)\right), \end{array}$$

where *I*_0_ is the intensity scalar, **P** is the 2 × 3 projection matrix which maps the three RGB signals to two signals, **N** is the normalization matrix such that the temporal mean signal is equal to the unit vector, **u**_*s*_ is the unit vector of the specular intensities while *s*(*t*) is the time varying specular intensity, **u**_*p*_ is the unit vector of the pulsatile intensities, and *p*(*t*) is the time-varying pulsatile intensity. The pulsatile amplitude is strongest in the green channel [24]. It follows that the projection matrix **P** is chosen as

(3) $$\begin{array}{c} P=\left[\begin{array}{ccc} 0 & 1 & -1\\ -2 & 1 & 1 \end{array}\right], \end{array}$$

which fulfills the orthogonality requirement. Next, *S*_1_(*t*) and *S*_2_(*t*) must be combined into one pulse signal. To do so, the standard deviations *σ*(*S*_2_) to *σ*(*S*_1_) are normalized as in equation (4).

(4) $$\begin{array}{c} h\left(t\right)=S_{1}\left(t\right)+\frac{\sigma \left(S_{1}\right)}{\sigma \left(S_{2}\right)\;}∙\sigma \left(S_{2}\right). \end{array}$$

When *S*_1_(*t*) and *S*_2_(*t*) are in phase, they push the amplitude of *h*(*t*) through constructive inference. If the two projected signals are in antiphase, they cancel each other. The underlying assumption here is that the specular part of the signal is rarely in phase with the pulsatile signal.

## 3. Results

### 3.1. Skin Temperature Measurement and Thermal Compensation

**Figure 3:**
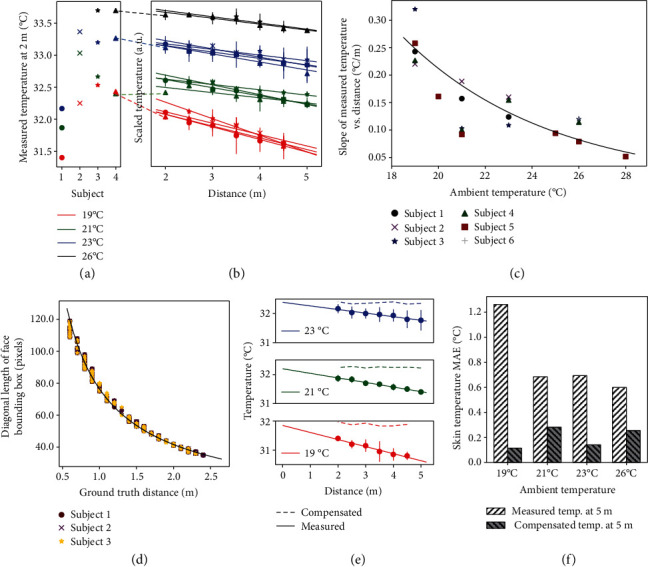
Thermal compensation of the IR camera for skin temperature measurement. (a) Measured skin temperature for subjects at 2 m. Different colors represent different ambient temperatures. (b) Skin temperature for subjects measured from 2 m to 5 m. Measured temperatures for each subject are translated vertically and grouped by ambient temperature. For the same subject and ambient temperature, measured temperatures in (a) represent the same data as the scaled temperatures in (b) at 2 m. (c) Effect of ambient temperature on the relationship between the measured temperature and the distance. (d) Dimensions of the face bounding box vs. subject's distance. (e) Measured and compensated skin temperatures for one subject. (f) Error analysis of measured skin temperatures and compensated skin temperatures for all subjects at 5 m. Data for 5 m at 19°C is imputed from the line of best fit.

To eliminate the need for a static, black body reference and thus enable a mobile platform, we propose a thermal compensation algorithm for the IR camera. Six subjects were recorded at distances ranging from 0.5 m to 5 m away from the camera and at ambient conditions ranging from 19°C to 28°C. Experimental results in Figures 3(a) and 3(b) show that skin temperature variations are affected both by the distance from the camera and the ambient temperature. At each ambient temperature, there is a linear relationship between the temperature and the distance. Note that Figures 3(a) and 3(b) show the subjects with recorded data from 2 m to 5 m, which is the intended operating distance for Dr. Spot. Appendix II in the Supplementary Information includes sample data for the other subjects.

The (negative) slopes for the relationship between the skin temperature and the distance at varying ambient temperatures are shown in Figure 3(c). Through inverse analysis of Figure 3(c), the compensated skin temperature (*T*_compensated_) can be determined from the IR camera measurement (*T*_IR_) using feedback from the ambient temperature (*T*_ambient_) and the subject's distance from camera, *D*.

(5) $$\begin{array}{c} T_{\text{compensated}}=T_{\text{IR}}+{T_{\text{ambient}}}^{-3.1}\left(1900∙D+3000\right)-0.17. \end{array}$$

To determine the subject's distance to the camera, we leverage the relationship between an object's bounding box and the object's distance from the camera [25]. Figure 3(d) shows the results from three different subjects with different genders and head sizes. The data from these subjects overlap with each other, which supports the estimation of the distance with face bounding box dimensions. These results show an inverse relationship between the subjects' distance and the diagonal length of their face bounding box, *L*.

(6) $$\begin{array}{c} D=140.22∙L^{-1.14}. \end{array}$$

A typical subject will not be facing directly at Dr. Spot. Rather, the subject's head may be tilted up/down with a pitch angle *θ*_pitch_ or tilted left/right with a yaw angle *θ*_yaw_. These parameters affect the face bounding box dimensions and thus the distance estimation. To determine *θ*_pitch_ and *θ*_yaw_, we use OpenCV's solvePnP method for pose estimation. Thus, instead of using the diagonal length, *L* in equation (5), we will use the corrected diagonal length *L*_corrected_.

(7) $$\begin{array}{c} L_{\text{corrected}}=\sqrt{{\left(\frac{{\text{width}}_{\text{face bounding box}}}{\cos\left(\theta_{\text{yaw}}\right)}\right)}^{2}+{\left(\frac{{\text{height}}_{\text{face bounding box}}}{\cos\left(\theta_{\text{pitch}}\right)}\right)}^{2}}. \end{array}$$

Substituting equations (6) and (7) into (5) results in the full equation for thermal compensation of IR camera measurements.

Figure 3(e) shows the measured and compensated temperatures for one subject. The compensated temperatures are calculated using the above-derived equations. Figure 3(f) shows the error analysis for the measured and compensated temperatures for all subjects at *5* m. The measured temperature has a maximum MAE of 1.3°C, which occurs at an ambient temperature of 19°C. The compensated temperature has a maximum MAE of 0.3°C, which occurs at an ambient temperature of 21°C. The proposed compensation method is able to account for the effects of distance and ambient temperature, significantly improving the accuracy skin temperature estimation.

### 3.2. Respiratory Rate Measurement

We first test our proposed method for RR measurement on one subject. Using these results, we select the optimal parameters for quick screening and continuous monitoring. Then, we set our method at these parameters and validate it on ten different subjects.

**Figure 4:**
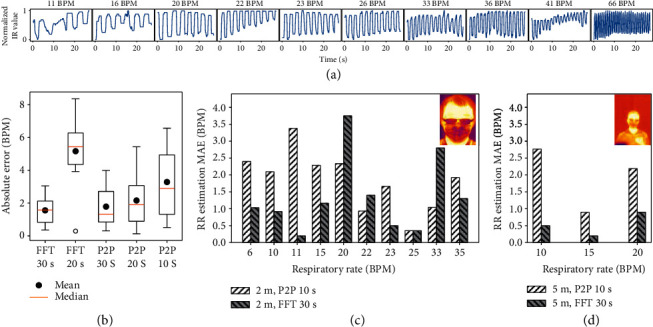
Respiratory rate estimation with the IR camera for subjects with facemasks. (a) Estimated RR waveforms for one subject after ten different levels of exercise. IR camera temperature readings are normalized from 0 to 1. (b) Error analysis of RR estimation for waveforms in (a) with peak-to-peak (P2P) and fast Fourier transform (FFT). Various window sizes are used to determine the best parameters for quick screening and continuous monitoring. (c) Error analysis of RR estimation for 10 subjects standing 2 m from the IR camera. P2P and FFT are used at the optimal parameters. (d) Error analysis of RR estimation for 2 subjects standing 5 m from the IR camera. P2P and FFT are used at the optimal parameters.

Figure 4(a) shows the test results for one subject after ten different levels of exercise. The raw breathing signal is obtained from the thermal readings of the facemask ROI. Inhalation and exhalation caused periodic troughs and peaks in the raw breathing signal, respectively. In the peak-to-peak (P2P) method for calculating RR, the average peak-to-peak values are computed across various window sizes to determine RR. In the fast Fourier transform (FFT) method for calculating RR, the RR is the frequency with the highest amplitude after applying FFT.

The window size is defined as the time window which is considered for one RR estimation. The length of the resulting vector *n* is determined by the sampling rate *f*_*s*_ of the camera and the window size *t*_*w*_ as *n* = *t*_*w*_∙*f*_*s*_. Choosing a sufficiently large measurement window is crucial for an accurate estimation since it controls the frequency resolution in the Fourier space. The frequency resolution *f*_*R*_ is defined as

(8) $$\begin{array}{c} f_{R}=\frac{f_{s}}{n}=\frac{1}{t_{w}}. \end{array}$$

Since *f*_*s*_ is constant for a given input stream, we can only choose *t*_*w*_. The IR camera on Dr. Spot has *f*_*s*_ = 30 Hz; setting *t*_*w*_ = 20 s results in a frequency resolution *f*_*R*_ = 0.05 Hz = 3 BPM. To ensure this minimum frequency resolution, the minimum window size for the FFT method is 20 s. However, this resolution constraint does not apply to the P2P method, which operates in the time domain. Rather, the minimum window size for the P2P method is constrained by the minimum RR to be measured. For a minimum RR of 6 BPM, the period is 10 s. To ensure that two peaks can be obtained from the waveform, two periods of the data must be captured. Thus, the minimum window size for the P2P method is 10 s.

To successfully screen for COVID-19 patients, Dr. Spot must be able to detect tachypnea (abnormally high RR). The maximum measurable RR can be determined from the Nyquist sampling theorem:

(9) $$\begin{array}{c} f_{s}\geq 2∙f_{\max}, \end{array}$$

where *f*_max_ is the maximum estimable frequency. Using the Nyquist theorem, the maximum estimable RR is 15 Hz or 900 BPM, which exceeds the maximum possible human RR.

To determine the optimal parameters for RR estimation, the RR is estimated for waveforms in Figure 4(a) at various window sizes and compared against ground truth sensor readings. These results are shown in Figure 4(b). The errors for both the P2P and FFT methods are not correlated with the subject's RR. At large window sizes (i.e., 30 s), the FFT method performs better; over a longer interval, noisy signals become attenuated. At smaller window sizes (i.e., 10 s), the P2P method performs better; obtaining more peak-to-peak measurements of a periodic signal does not greatly increase accuracy.

The most accurate method is using FFT at the largest window size of 30 s, which results in average RR error 1.6 BPM. The fastest method is using P2P at the smallest window size of 10 seconds, which results in an average RR error of 3.3 BPM. Since the P2P method performs better at smaller measurement windows, it is used for rapid screening of patients. Conversely, since the FFT method is more accurate but requires larger measurement windows, it is used for continuous monitoring of patients.

Having determined the optimal parameters, we validate our method on 32 waveforms recorded on ten healthy subjects at a distance of 2 m. Their respiratory rates ranged from 6 BPM to 35 BPM. Abnormal respiratory rates were simulated by asking subjects to follow a predetermined, coached breathing patterns displayed to participants in real time. Appendix III in the Supplementory Information shows all 32 respiratory waveforms recorded from the 10 subjects using Dr. Spot.  Figure 4(c) shows the error analysis of these 10 subjects. Continuous monitoring using FFT with a window size of 30 s is most accurate, with MAE = 1.6 BPM. Quick screening with P2P is acceptable, with MAE = 2.1 BPM.

The facemask region forms a large region of interest on a subject. Since the RR is obtained from the facemask region, the proposed RR method can work at larger distances. We validate our method 6 waveforms recorded on two healthy subjects at a distance of 5 m. Their respiratory rates ranged from 10 BPM to 20 BPM. Continuous monitoring using FFT with a window size of 30 s remains accurate, with MAE = 0.6 BPM. Quick screening with P2P remains acceptable, with MAE = 2.0 BPM.

### 3.3. Heart Rate Measurement

**Figure 5:**
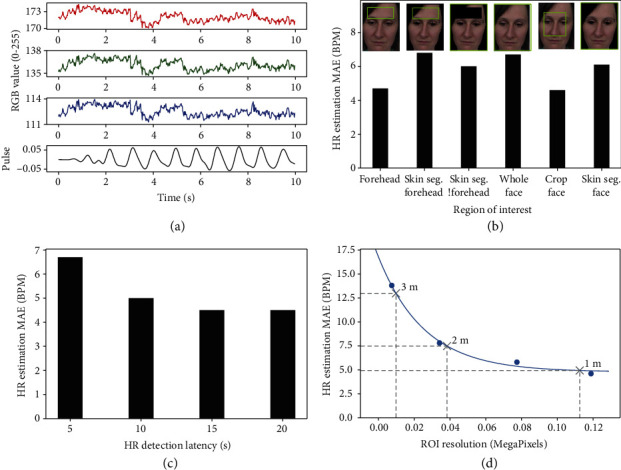
Heart rate estimation with monochrome cameras. (a) RGB signals captured from the subject's forehead in a normal ambient lighting condition. Pulse signal determined from RGB signals using the POS method. (b) Effect of region of interest (ROI) on HR estimation accuracy. Skin. Seg. means skin segmentation. !forehead means excluding forehead. (c) Effect of detection latency (amount of time spent capturing data before estimating HR) on HR estimation accuracy. (d) Effect of ROI resolution on HR estimation accuracy. The ROI resolution (i.e., forehead resolution) captured by a 5 MP monochrome camera at 1 m, 2 m, and 3 m is displayed.

HR is determined using the POS method, which was selected for its high accuracy and real-time performance (a more detailed discussion is presented in Supplementary Information Appendix IV).  Figure 5(a) shows sample RGB signals captured by the monochrome cameras in an arbitrary lighting condition and the resulting HR pulse calculated using the POS method. The estimated HR is 66 BPM, while the ground truth is 63 BPM. This value is less than 100.0 BPM, resulting in a negative detection for tachycardia. rPPG with the POS method has been experimentally validated by de Haan and Van Least in a well-controlled environment with uniform lighting condition [23]; it is not further validated in subjects wearing a mask while maintaining social distancing. Rather, we focus on characterizing the POS method at various parameters to optimize HR estimation for Dr. Spot.

Various ROIs can be used to estimate HR, such as the face or the forehead. These ROIs can be cropped using object detection methods or segmented using skin segmentation methods.  Figure 5(b) shows the POS method evaluated for various ROIs on the UBFC-rPPG dataset. The forehead and cropped face are the most accurate for HR estimation. However, Dr. Spot must estimate HR for subjects wearing facemasks in a hospital triage environment. Since the subjects will have their faces covered, we select the forehead as the ROI for HR estimation.

HR can be estimated after various latencies; a latency of *n* seconds means that the subject is recorded for *n* seconds before an HR estimation is made.  Figure 5(c) shows the POS method evaluated for 11 subjects at various latencies. The POS method produces more accurate HR estimations with more recorded data. However, there is only a 10% difference between the HR estimation error for a 10 s latency and 20 s latency. As discussed in Section 3.2, RR quick screening requires 10 s. Thus, we use a HR detection latency of 10 s so that both HR and RR and be determined in the same amount of time.

The distance of a subject from the camera affects their ROI resolution.  Figure 5(d) shows the POS method evaluated at various ROI resolutions, with the forehead as the ROI. The HR estimation error decreases exponentially with a decreasing subject distance. For a subject at 2 m, the HR estimation MAE is 7.5 BPM.

## 4. Discussion

In this paper, we presented algorithms to enable a mobile, robotic platform to monitor vital signs (skin temperature, heart rate, and respiratory rate) using one IR camera and three monochrome cameras without the need of standardized ambient conditions and fixed measuring distance. These algorithms are scalable through the use of commercial camera systems and can enable a socially distanced healthcare worker to easily screen for abnormal vital signs within the first 10 seconds of a patient encounter. Such a system is innovative and novel because it removes key boundary conditions traditionally used with IR cameras and provides an implementation pathway that allows healthcare systems to adopt contactless systems for vital sign screening and continuous monitoring.

IR cameras have previously been used for skin temperature measurement and fever screening, yet these systems require a fixed camera and highly regulated ambient conditions [13]. The patients undergoing screening for fever must stand at a specified position and directly face the camera to ensure an accurate reading. While these requirements may be acceptable in some situations, healthcare settings where a contactless system may have high impact may not be able to standardize conditions to permit existing IR systems to be adopted. Our proposed method for thermal compensation measures the skin temperature without using a static black body, thus enabling a mobile platform which increases opportunities for deployment in nontraditional settings like emergency departments and field hospitals. This results in a robust system that is able to automatically correct for the ambient temperature and distance to the camera to provide accurate skin temperature readings.

We presented a novel method for measuring the respiratory rate that relies on periodic temperature contrasts in the facemask region of IR images. This method is particularly applicable during the COVID-19 pandemic, where facemask mandates have been enacted, especially in indoor settings like hospitals. While mask mandates may change based on local COVID-19 infection rates and political stances, most hospital settings will likely have enduring facemask requirements to prevent disease transmission and protect healthcare workers. In this setting, a contactless system which leverages the use of facemasks to calculate the respiratory rate will continue to be applicable.

Recall that the POS method was used and adapted in the estimation of the heart rate. An important property of the POS method is that it utilizes the relative pulsatile amplitudes in the monochrome camera channels to differentiate variations in blood volume from variations from other sources such as motion. However, since the rPPG methods rely on images from monochrome cameras, the HR estimation is highly sensitive to factors such as lighting conditions and subject demographics [26]. Further work is required to create more robust rPPG methods. Though Dr. Spot is able to monitor skin temperature, HR, and RR, verification was only performed on limited numbers of healthy volunteers that approximated high HR and RR through vigorous exercise. More testing is required to further verify the accuracy of the proposed methods. Lastly, Dr. Spot is potentially capable of monitoring SpO_2_. However, it would require tremendous amounts of experimentation to calibrate the ambient lighting conditions as well as subject skin tone correction, which is beyond the scope of this work.

## 5. Conclusion

We developed a camera system consisting of one IR camera and three monochrome cameras to reliably facilitate contactless acquisition of vital sign parameters central to triaging and managing individuals with COVID-19 disease. This camera system was mounted on a teleoperated robot, Dr. Spot, which can successfully and reliably deliver vital sign measurements while navigating in complex clinical environments and maintaining social distancing. In the COVID-19 pandemic, deploying Dr. Spot helps conserve PPE, curbs transmission of infection, and helps clinical detect key vital sign abnormalities.

## Data Availability

The authors confirm that the data supporting the findings of this study are available within the article and its supplementary materials.

## Supplementary Materials

Supplementary Materials Figure S1: floor plan of the COVID-19 Triage Tent at Brigham and Women's Hospital outside the emergency department. Figure S2: experimental validation of skin temperature compensation for a subject from 0.6 m to 3.0 m. Figure S3: respiratory rate validation with 10 subjects using the proposed method in which the IR camera temperature readings are normalized from 0 to 1. Figure S4: (A) heart rate estimation error and (B) frame rate of various rPPG methods evaluated based on the UBFC-rPPG dataset. Figure S5: heart rate estimation error using the modified POS method evaluated based on the UBFC-rPPG dataset [27].
