# Supplementary material for: Mobile Robotic Platform for Contactless Vital Sign Monitoring
Source: Cyborg Bionic Syst. 2022 Apr 30;2022:9780497. doi: 10.34133/2022/9780497 (PMC9096356; doi:10.34133/2022/9780497)
Supplement: Supplementary Materials — Figure S1: floor plan of the COVID-19 Triage Tent at Brigham and Women's Hospital outside the emergency department. Figure S2: experimental validation of skin temperature compensation for a subject from 0.6 m to 3.0 m. Figure S3: respiratory rate validation with 10 subjects using the proposed method in which the IR camera temperature readings are normalized from 0 to 1. Figure S4: (A) heart rate estimation error and (B) frame rate of various rPPG methods evaluated based on the UBFC-rPPG dataset. Figure S5: heart rate estimation error using the modified POS method evaluated based on the UBFC-rPPG dataset [27]. [file 9780497.f1.zip › Supplementary information_final.docx]

**Appendix I:**


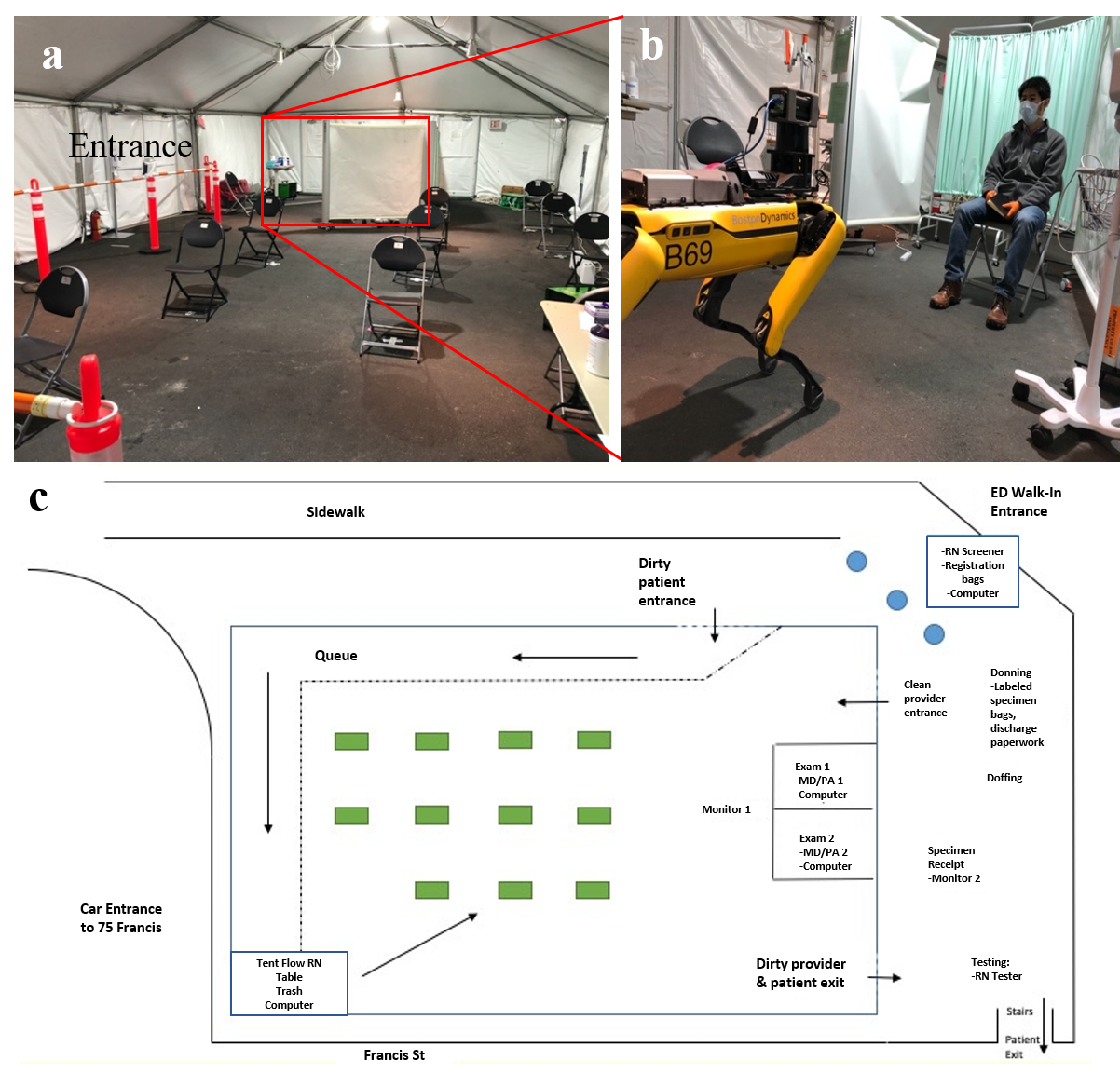


Figure S1. Floor plan of the COVID-19 Triage Tent at Brigham and Women’s Hospital outside the emergency department.

**Appendix II:**

Figure S2 shows experimental results of the thermal compensation method on a subject for distances less than 3 *m*. The temperature compensation is able to account for the ambient conditions and the distance of the subject to camera. At a distance of 3 *m*, the measured skin temperature has an error of 1.1 ***°****C*, while the compensated skin temperature has an error of 0.2 ***°****C*.

Figure S2. Experimental validation of skin temperature compensation for a subject from 0.6 *m* to 3.0 *m.*

**Appendix III:**

Figure S3. Respiratory rate validation with 10 Subjects using the proposed method in which the IR camera temperature readings are normalized from 0 to 1.

**Appendix IV:**

This appendix compares different rPPPG algorithms in terms of their accuracy and computational speed on the UBFC-rPPG dataset (Figure S4). The algorithms examined are the Plane-orthogonal-to-skin (POS), the chrominance algorithm (CHROM), pulse blood volume (PBV) and the adaptive pulse blood volume (APBV) method. The PBV and APBV methods are usually evaluated on closed datasets using the signal-to-noise ratio (SNR) as the evaluation metric [8] [22]. However, when using estimation accuracy as the evaluation metric, we find that the PBV and APBV methods are not suitable to calculate HR (Figure S4A). The POS method shows the best results in terms of error, although we were not able to achieve as low error rates as shown in previous studies with any of the methods [26]. On the other, hand, the POS method is significantly slower than the other methods. CHROM, APBV and PBV can achieve a frame rate $> 50$ FPS while POS achieves 7 FPS for a $20s$ window at $30$ FOS camera frame rate i.e. $600$ processing samples (Figure S4B). The difference is due to the overlap adding in the POS algorithm which increases the time complexity depending on the window length. Multithreading can be implemented on the main processing unit to overcome this issue since we usually calculate the pulse signal every 5s, which is sufficient time to calculate the pulse signal while streaming the data. The APBV and PBV performance issue is caused by not calibrating the PBV vector. Since we aim for a mobile system, it is not feasible to calibrate the upbv vector when the lighting condition is changing which makes these two methods unusable. Although CHROM is faster than POS, we choose to continue with POS since it has approximately $35\%$ and $40\%$ lower MAE and RMSE respectively. Figure S5 shows the error analysis using the POS method based-on eleven human subjects in a normal ambient lighting condition at least three measurements from each subject. The MAE and RMSE is 4.5 BPM and 6.1 BPM, respectively. The subjects are of various skin tones, gender, and age between 18 years old and 30 years old.


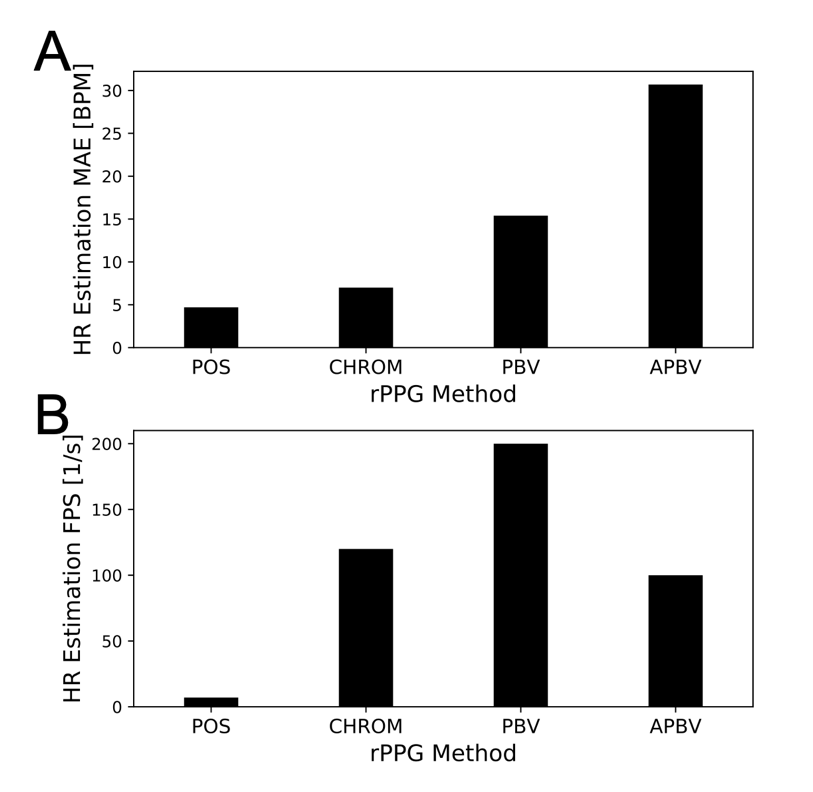


Figure S4. (A) Heart rate estimation error and (B) frame rate of various rPPG methods evaluated based-on the UBFC-rPPG Dataset


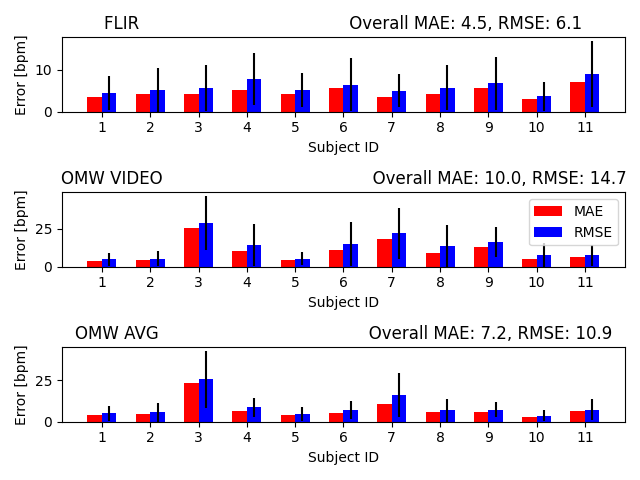


Figure S5. Heart rate estimation error using the modified POS method evaluated based-on
