# Supplementary figures and images for: Mobile Robotic Platform for Contactless Vital Sign Monitoring
Source: Cyborg Bionic Syst. 2022 Apr 30;2022:9780497. doi: 10.34133/2022/9780497 (PMC9096356; doi:10.34133/2022/9780497)

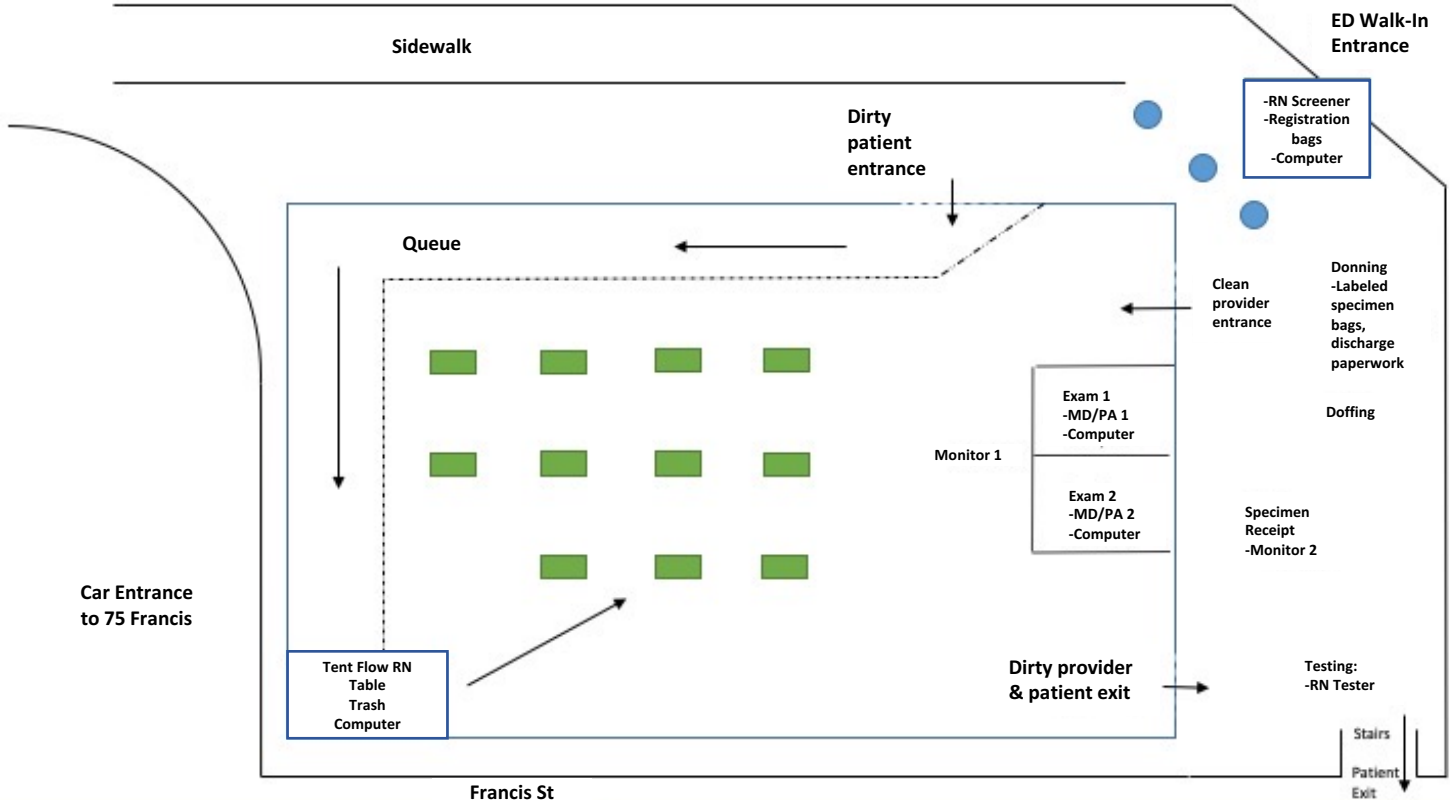

Supplement: Supplementary Materials — Figure S1: floor plan of the COVID-19 Triage Tent at Brigham and Women's Hospital outside the emergency department. Figure S2: experimental validation of skin temperature compensation for a subject from 0.6 m to 3.0 m. Figure S3: respiratory rate validation with 10 subjects using the proposed method in which the IR camera temperature readings are normalized from 0 to 1. Figure S4: (A) heart rate estimation error and (B) frame rate of various rPPG methods evaluated based on the UBFC-rPPG dataset. Figure S5: heart rate estimation error using the modified POS method evaluated based on the UBFC-rPPG dataset [27]. [file 9780497.f1.zip › Figure S1.pdf]

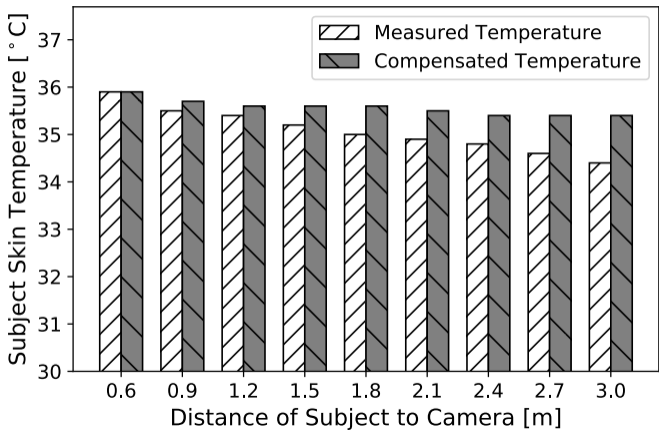

Supplement: Supplementary Materials — Figure S1: floor plan of the COVID-19 Triage Tent at Brigham and Women's Hospital outside the emergency department. Figure S2: experimental validation of skin temperature compensation for a subject from 0.6 m to 3.0 m. Figure S3: respiratory rate validation with 10 subjects using the proposed method in which the IR camera temperature readings are normalized from 0 to 1. Figure S4: (A) heart rate estimation error and (B) frame rate of various rPPG methods evaluated based on the UBFC-rPPG dataset. Figure S5: heart rate estimation error using the modified POS method evaluated based on the UBFC-rPPG dataset [27]. [file 9780497.f1.zip › Figure S2.pdf]

Normalized IR Value

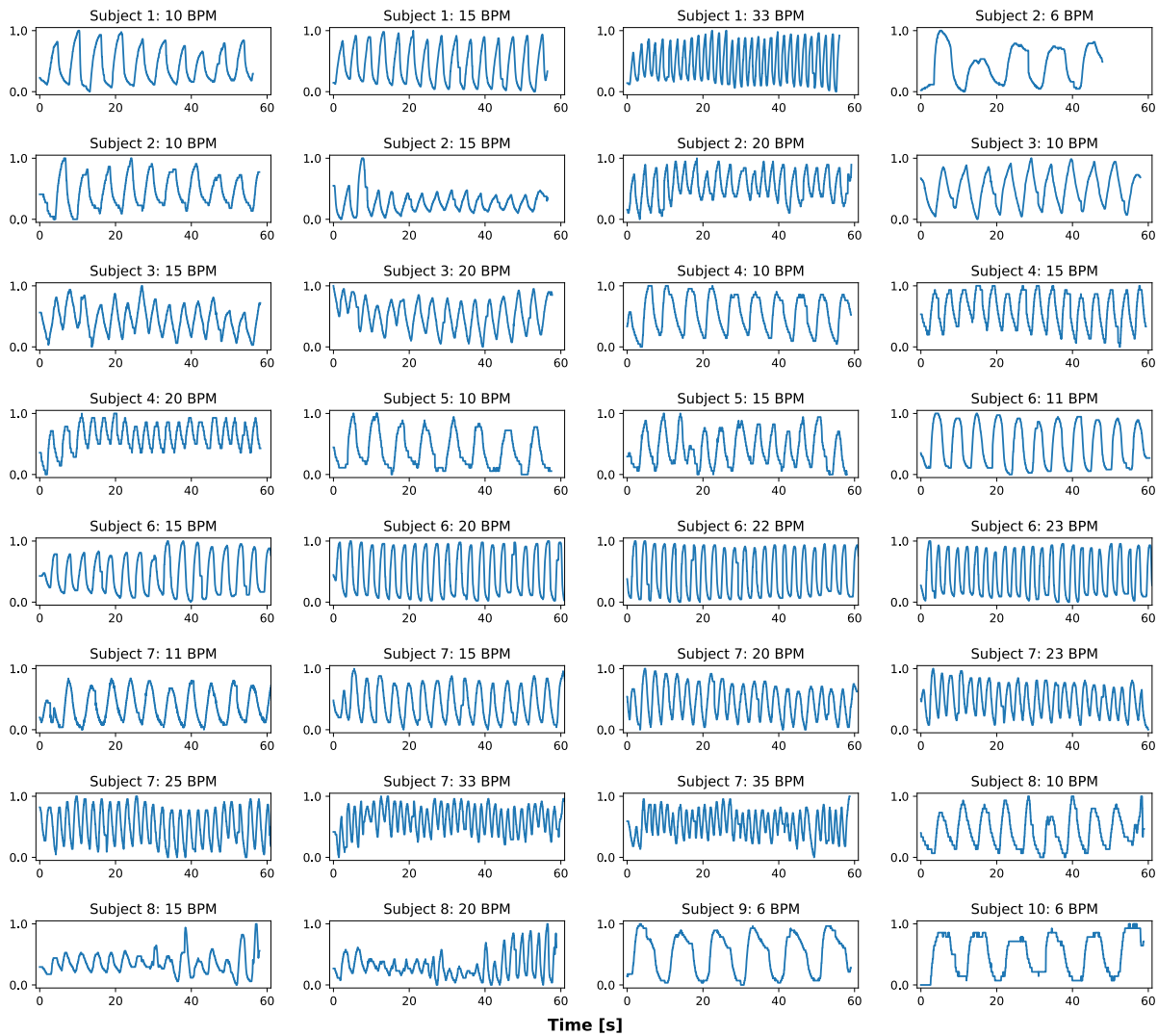

Supplement: Supplementary Materials — Figure S1: floor plan of the COVID-19 Triage Tent at Brigham and Women's Hospital outside the emergency department. Figure S2: experimental validation of skin temperature compensation for a subject from 0.6 m to 3.0 m. Figure S3: respiratory rate validation with 10 subjects using the proposed method in which the IR camera temperature readings are normalized from 0 to 1. Figure S4: (A) heart rate estimation error and (B) frame rate of various rPPG methods evaluated based on the UBFC-rPPG dataset. Figure S5: heart rate estimation error using the modified POS method evaluated based on the UBFC-rPPG dataset [27]. [file 9780497.f1.zip › Figure S3.pdf]

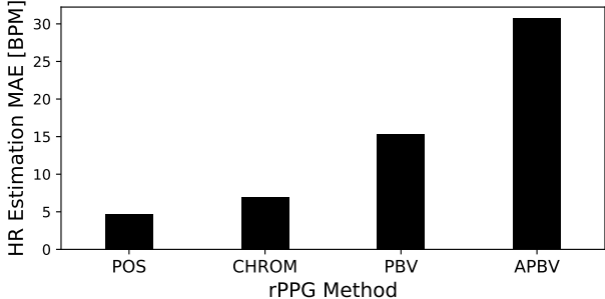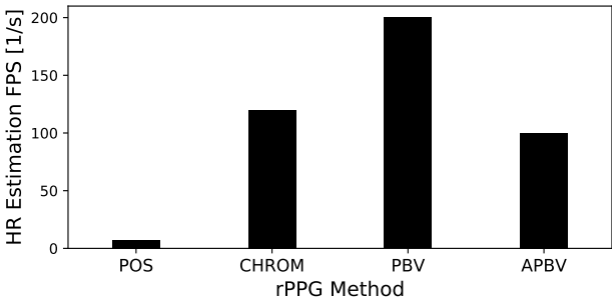

Supplement: Supplementary Materials — Figure S1: floor plan of the COVID-19 Triage Tent at Brigham and Women's Hospital outside the emergency department. Figure S2: experimental validation of skin temperature compensation for a subject from 0.6 m to 3.0 m. Figure S3: respiratory rate validation with 10 subjects using the proposed method in which the IR camera temperature readings are normalized from 0 to 1. Figure S4: (A) heart rate estimation error and (B) frame rate of various rPPG methods evaluated based on the UBFC-rPPG dataset. Figure S5: heart rate estimation error using the modified POS method evaluated based on the UBFC-rPPG dataset [27]. [file 9780497.f1.zip › Figure S4.pdf]

FLIR

Overall MAE: 4.5, RMSE: 6.1

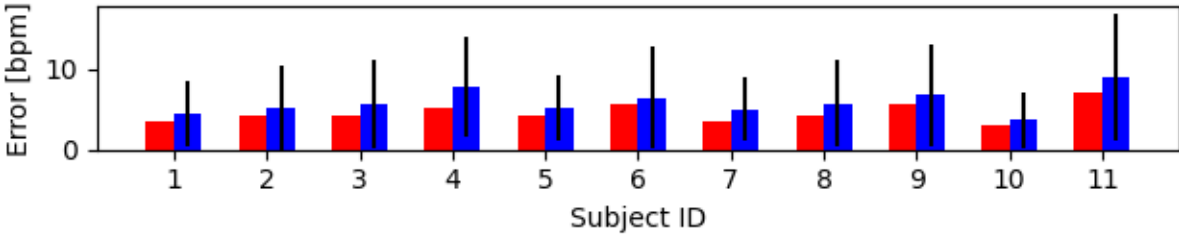

Supplement: Supplementary Materials — Figure S1: floor plan of the COVID-19 Triage Tent at Brigham and Women's Hospital outside the emergency department. Figure S2: experimental validation of skin temperature compensation for a subject from 0.6 m to 3.0 m. Figure S3: respiratory rate validation with 10 subjects using the proposed method in which the IR camera temperature readings are normalized from 0 to 1. Figure S4: (A) heart rate estimation error and (B) frame rate of various rPPG methods evaluated based on the UBFC-rPPG dataset. Figure S5: heart rate estimation error using the modified POS method evaluated based on the UBFC-rPPG dataset [27]. [file 9780497.f1.zip › Figure S5.pdf]
